# Incidence of intrapartum stillbirth and associated risk factors in tertiary care setting of Nepal: a case-control study

**DOI:** 10.1186/s12978-016-0226-9

**Published:** 2016-08-31

**Authors:** Ashish KC, Johan Wrammert, Uwe Ewald, Robert B. Clark, Jageshwor Gautam, Gehanath Baral, Kedar P. Baral, Mats Målqvist

**Affiliations:** 1 International Maternal and Child Health, Department of Women’s and Children’s Health, University Hospital, SE-751 85 Uppsala, Sweden; 2 United Nation’s Children’s Fund, Nepal Country Office, UN House, Pulchowk, Nepal; 3 Latter-day Saint Charities, Salt Lake City, UT USA; 4 Paropakar Maternity and Women’s Hospital, Thapathali, Nepal; 5 Patan Academy of Health Sciences, Lalitpur, Nepal

## Abstract

**Background:**

Each year, 1.2 million intrapartum stillbirths occur globally. In Nepal, about 50 % of the total number of stillbirths occur during the intrapartum period. An understanding of the risk factors associated with intrapartum stillbirth will facilitate the development of preventative strategies to reduce the associated burden of death. This study was conducted in a tertiary-care setting with the aim to identify risk factors associated with intrapartum stillbirth.

**Methods:**

A case–control study was completed from July 2012 to September 2013. All women who had an intrapartum stillbirth during the study period were included as cases, and 20 % of women with live births were randomly selected upon admission to create the referent population. Relevant information was retrieved from clinical records for case and referent women. In addition, interviews were completed with each woman to determine their demographic and obstetric history.

**Results:**

During the study period, 4,476 women were enrolled as referents and 136 women had intrapartum stillbirths. The following factors were found to be associated with an increased risk for intrapartum stillbirth: poor familial wealth quintile (Adj OR 1.8, 95 % CI-1.1–3.4); less maternal education (Adj OR, 3.2 95 % CI-1.8–5.5); lack of antenatal care (Adj OR, 4.8 95 % CI 3.2–7.2); antepartum hemorrhage (Adj OR 2.1, 95 % CI 1.1–4.2); multiple births (Adj. OR-3.0, 95 % CI- 1.9–5.4); obstetric complication during labor (Adj. OR 4.5, 95 % CI-2.9–6.9); lack of fetal heart rate monitoring per protocol (Adj. OR-1.9, 95 % CI 1.5–2.4); lack of partogram use (Adj. OR-2.1, 95 % CI 1.1–4.1); small-for-gestational age (Adj. OR-1.8, 95 % CI-1.2–1.7); preterm birth (Adj. OR-5.4, 95 % CI 3.5–8.2); and being born preterm with a small-for-gestational age (Adj. OR-9.0, 95 % CI 7.3–15.5).

**Conclusion:**

Being born preterm with a small-for-gestational age was associated with the highest risk for intrapartum stillbirth. Inadequate fetal heart rate monitoring and partogram use are preventable risk factors associated with intrapartum stillbirth; by increasing adherence to these interventions the risk of intrapartum stillbirth can be reduced. The association of the lack of appropriate antenatal care with intrapartum stillbirth indicates that quality antenatal care may improve fetal health and outcomes.

**Trial registration:**

ISRCTN97846009

## Plain English summary

Stillbirth during labor (intrapartum stillbirth) is a global problem. In Nepal, more than half of stillbirth takes place during labor. Identifying risk factors associated with intrapartum stillbirth may provide evidence to develop preventive strategies.

A research study was conducted at a tertiary level hospital in Nepal, Paropakar Maternity and Women’s Hospital, to identify risk factors associated with stillbirth occurring during labor. The study was conducted from July 2012 to September 2013. Relevant information was retrieved from clinical records and interviews were completed with each woman to determine their demographic and obstetric history.

The study found the woman from women from poorer families had higher risk for intrapartum stillbirth. Similarly, uneducated woman without antenatal care, woman who had obstetric complications during pregnancy and labor had associated risk of intrapartum stillbirth. Woman whose labor progress was not adequately assessed using partogram and fetal heart rate not monitored as per standard had risk for intrapartum stillbirth. Further results show that small and premature babies had the highest risk of stillbirth.

Conclusion: Being born prematurely and small had the highest risk for intrapartum stillbirth. Inadequate fetal heart rate monitoring and partogram use are preventable risk factors associated with intrapartum stillbirth; by increasing compliance to these interventions the risk of intrapartum stillbirth might be reduced.

## Background

In many societies, and on the worldwide policy agenda, stillbirths are not accounted for despite the fact that each stillbirth is a tragedy for mothers and their families [1, 2]. Although the global burden of disease measurement does not include stillbirths, 2.65 million stillbirths (delivered at ≥ 1000 g or ≥ 28 weeks of gestation) occur annually [3, 4]. Ninety eight percent of third trimester stillbirths occur in low- and middle-income countries, with more than three quarters of them in South Asia and sub-Saharan Africa [1]. The period surrounding labor and delivery represents the time of highest risk, when 45 % of all stillbirths occur [1]. With improvements in the quality of intrapartum care in high-income countries, most of these stillbirths have been averted [5–7]. Of the total number of stillbirths that occur in South Asia, 57 % are during the intrapartum period [1]. Variation in the rate of intrapartum stillbirth within countries is wide and depends upon the readiness of health facilities to provide intrapartum care, as well as the preparedness of the birth attendant for each delivery. Identifying risk factors for intrapartum stillbirth, and delays in the quality of care provided, is critical to identify appropriate interventions to reduce intrapartum stillbirth.

Maternal health is closely related to newborn health, and there are a number of risk factors for poor maternal health that have been linked to poor fetal outcomes [8]. Two systematic reviews done by Lawn et al. [9] and Di Mari et al. [10], have revealed several risk factors for third trimester stillbirth including: adolescent or elderly pregnancy; grand multi-parity; poor maternal nutrition, such as low body mass index or severe anemia; maternal medical conditions during pregnancy; exposure to toxic substances, such as tobacco, use of biomass for cooking or environmental toxins; and socio-economic deprivation, i.e., poor access to healthcare services during pregnancy, either due financial barriers or inadequate access to information [9, 10].

However, there are few studies from low-income countries identifying preventable risk factors for intrapartum stillbirth, especially in settings where obstetric care is readily available [11]. Population-based studies conducted in low- and middle-income countries have shown that obstetric complications during the intrapartum period, such as preeclampsia, fetal mal-presentation, prolonged labor, preterm delivery, or cesarean section, are associated with intrapartum stillbirth [12–14]. A population-based study in Ghana revealed that poverty constituted the highest risk for intrapartum stillbirth, and furthermore, that this risk was not influenced by health care utilization [15]. A systematic review by Lawn et al. [16] examining risk factors for intrapartum stillbirth indicated that intrapartum stillbirth is preventable, as 25–67 % are primarily due to preventable intrapartum complications, such as prolonged labor [16]. If effective interventions are implemented at the facility level, it is likely that a number of these stillbirths could be prevented. Similarly, hospital-based audits in several low-income countries have demonstrated that more than 25 % of intrapartum stillbirths could have been prevented with improved obstetric care [17–20].

Identifying preventable risk factors for intrapartum stillbirth in low-income countries like Nepal is important as the current rate of intrapartum stillbirth is 13.4 per thousand deliveries, and these stillbirths account for 55 % of the total stillbirth rate [1]. Over the past 10 years, there has been a slow decline in the number of intrapartum stillbirths, accompanied by an increase in institutional delivery, with more than 60 % of Nepali women now delivering at a health facility [4, 21–25]. Thus, understanding the risk factors for intrapartum stillbirth in the health facility setting, and developing strategies to reduce the number of preventable risk factors is critical.

The aim of this study was to identify preventable risk factors associated with intrapartum stillbirth in a tertiary care setting in Nepal.

## Methods

### Study design

We used a case–control study design nested within a larger prospective cohort study. All women delivering in the hospital were included in the source population, from which 20 % were randomly selected to be in the referent population. This 20 %, i.e. the referent population, was selected at the time of their admission to the hospital using a lottery technique. All women experiencing an intrapartum stillbirth during the study period were included in the case population. Any antepartum stillbirth occurring in the referent population was excluded from this study; while any intrapartum stillbirth that occurred in the referent population was excluded from that population and re-categorized for inclusion in the case population. The sample size of this study was based on calculations used in the larger prospective cohort study, which aimed to detect a 20 % reduction in perinatal mortality with a statistical power of 80 % and a level of significance of 5 %.

Ethical approval for this study was obtained from the Institutional Review Committee at Paropakar Maternity and Women’s Hospital, the Nepal Health Research Council (reg. 37/2012) and from Uppsala University (Sweden) (dnr. 2012/267), as part of a larger cohort study evaluating the impact of a Helping Babies Breathe quality improvement cycle on perinatal mortality [26]. The study was registered as clinical trial under the registration number: ISRCTN 97846009. The protocol for the study can be accessed from http://bmcpediatr.biomedcentral.com/articles/10.1186/1471-2431-12-159. Written consent was received from each of the study participants prior to their inclusion.

### Study setting

We conducted this study at Paropakar Maternity and Women’s Hospital; a tertiary, government-funded hospital located in Kathmandu, Nepal. The hospital has about 22,000 deliveries each year, with an institutional intrapartum stillbirth rate of nine per thousand births [27]. The hospital provides comprehensive maternal care services delivered by obstetricians, medical doctors and nurse midwives. The clinical protocol designed for the assessment of women coming to the hospital for delivery and completed upon their admission includes: ascertainment of gestational age and assessment of fetal heart sound, obstetric complication, and stage of labor. Based on these assessments, and the risk category assigned, each woman was then transferred to one of three different delivery units for intrapartum care. This study was conducted over a period of 15 months from July 2012 to September 2013.

### Participants

In this study, all intrapartum stillbirths occurring after hospital admission, i.e. among women who were in labor with detectable fetal heart sounds upon admission, were included as cases. Women that had fetal death at admission, i.e. absence of fetal heart sounds, were excluded. Similarly, antepartum stillbirths occurring prior to the onset of labor were also excluded from the case population. All women who were randomly selected to be in the referent population were included in this population if they had a live birth. Antepartum stillbirths were also excluded from the referent population.

### Data collection

A surveillance system was set up by recruiting 12 female surveillance officers to be stationed in the admission, delivery, and postnatal units. Any woman admitted to the hospital for delivery was marked in the surveillance registry. From this sampling frame, study participants were randomly selected using a lottery technique. Specifically, an opaque jar with 100 balls was kept in the admission unit, of which 80 were white and 20 were yellow. Upon each admission, a ball was drawn from the opaque jar; if a yellow ball was selected, the woman was enrolled into the study as part of the referent population. When a woman was selected as part of the referent population, she was tracked from the point of admission through her discharge to assess labor progression and birth outcomes.

The surveillance team in the delivery unit observed all deliveries together with referent population. When intrapartum stillbirth occurred in the delivery unit, the surveillance officers enrolled the woman with intrapartum stillbirth into the study as part of the case population. Woman in case population was tracked from delivery through her discharge. Women in case population (intrapartum stillbirth) had also received the same rigorous observations during labor and birth as the women in referent population. Information collected for women in case and referent population remained the same. For both the referent and case populations, information on parity, previous obstetric and medical history, care received during the current pregnancy, obstetric or medical complications during this pregnancy, and intrapartum care was retrieved from clinical record forms. Surveillance team members conducted interviews at the time of discharge with each woman using a questionnaire designed to assess the woman’s social, demographic and household information.

After receiving the completed clinical record and interview forms from the surveillance officers, a research manager checked each form for completeness. Additionally, 10 % of clinical record forms were checked against the primary data source to ensure data accuracy. Data entry officers reassessed the completeness of all forms, recoded open-ended response questions, and entered the data from each checked form into a CS-Pro database. To prevent data loss, indexing of all collected forms was performed. After data entry and data cleaning in the CS-Pro database was completed, the dataset was exported to SPSS 17 for data analysis.

### Variables

*Intrapartum stillbirth* was defined as the delivery of any fetus after 22 weeks of gestation, or with a birth weight more than 500 g, who had detectable fetal heart sounds upon admission, but died during the intrapartum period and thus had an Apgar score of 0 at 1 and 5 min, without signs of maceration. Intrapartum stillbirth cases were retrieved from the clinical journal [1].

*Antepartum stillbirth* was defined as the delivery of any non-viable fetus after 22 weeks of gestation, or with a birth weight more than 500 g, with an Apgar score of 0 at 1 and 5 min and signs of maceration, or absent fetal heart sound before the initiation of labor [1].

*Maternal age* was categorized into five-year intervals.

*Maternal education* was categorized into two groups as women who had 5 years or less than 5 years of education (primary education), and those who had six or more years of education (secondary education or higher).

*Ethnicity* was categorized into groups according to the social caste system within Nepal [28] as most advantaged (Brahmin/Chettri); relatively advantaged Janajatis (Newar, Gurung and Thakali); relatively disadvantaged Janajatis; relatively disadvantaged non-Dalit; most disadvantaged (Dalit and Muslim).

*Wealth index* was used as a measure of socioeconomic position and constructed according to the nationally representative health surveys (Demographic Health Surveys), to compare socioeconomic inequalities [29, 30]. During interviews with each mother, data was collected on ownership of durable assets (e.g. car, refigerator, bicycle, radio, television), housing characteristics (e.g. number of rooms, dwelling floor and roof materials, toilet facilities), and access to services (e.g. electricity supply, drinking water source). Using the scores from the first principal component analysis, a wealth index (asset index) was constructed. Based on the value of this index, individuals were sorted and population quintiles were established using cut-off values. These quintiles were then ranked from bottom to top as poorest, poorer, middle, richer and richest [31].

*Antenatal care attendance* was determined based on whether a mother attended any antenatal care (ANC) visits during which she received a clinical examination, counseling, and medication (if needed) from a skilled provider as per guidelines. ANC was categorized into two groups as those who attended at least one ANC visit, and those who did not receive any ANC at all.

*Parity* was based on the number of times a woman had previously given birth after the age of viability, i.e. 22 weeks, including both live and still births [32]. Parity was categorized into three groups including primiparous, multiparous (1–2) or multiparous (3 or more).

*Previous stillbirth* was categorized as whether the women had any stillbirth in a previous pregnancy(s), or not.

*Antepartum hemorrhage* was defined as vaginal bleeding prior to the onset of labor. This was categorized into two groups as those having any antepartum hemorrhage, or none.

*Hypertensive disorder of pregnancy* was defined as a maternal diastolic blood pressure of 90 mmHg or more in two consecutive assessments, which were at least four hours apart, during pregnancy. This was categorized as those having the condition in the current pregnancy, or not.

*Medical complication during pregnancy* was considered present in women having diabetes mellitus, severe anemia (Hb <7 gm/L), or epilepsy during the current pregnancy.

*Multiple birth* included women pregnant with more than one fetus.

*Obstetric complication during delivery* was defined as any complication that a woman had during the intrapartum period [33], including:

*Hypertensive disorder*: Classified by maternal diastolic blood pressure greater than or equal to 90 mmHg in two separate recordings
*Mal-presentation*: Presentation of the fetus in any position besides vertex, i.e. with the top of the head appearing first
*Prolonged labor*: Cervical dilation that does not move beyond 4 cm after eight hours of regular contractions, or cervical dilation lying to the right of the alert line on the partogram; and
*Prolapsed cord*: Characterized by the presence of the umbilical cord in the birth canal below the fetal presenting part, or at the vagina following the rupture of membranes.

*Fetal Heart Rate Monitoring (FHRM) per protocol* was considered adequate when the fetal heart rate was measured at least every half an hour using the auscultation technique, during the intrapartum period. Any labor in which fetal heart rate was not monitored within every half an hour was categorized as non-adherent to protocol.

*Adherence to partogram use* was considered adequate when the partogram was used, i.e. filled in for the progress of cervical dilation and descent of the head, every half an hour to assess the progression of labor. Any case where labor progression was not adequately monitored using the partogram was categorized as non-adherent.

*Gestational age of the baby* was categorized into two groups as preterm or term according to the following defintitions:

*Preterm birth*: which included babies born before 37 completed weeks of gestation, estimated by the date of the mother’s last menstrual period or based on clinical examination of the newborn
*Term birth*: which included babies who were born at, or after, 37 completed weeks of gestation, estimated by the mother’s last menstrual period or based on clinical examination of the newborn.

*Weight for gestational age at birth* was categorized into two groups as small- or appropriate- for gestational age according to the following definitions:

*Small-for-gestational age (SGA)*: which included babies whose birth weight was less than the 10^th^ percentile according to the appropriate gestational age and sex-specific reference population standards [34]
*Appropriate for gestational age (AGA)*: which included babies whose birth weight was greater than or equal to the 10^th^ percentile according to the appropriate gestational age and sex-specific reference population standards [34].

### Statistical analysis

The comparison of demographic and obstetric characteristics of the women in the referent and case populations was performed using Pearson’s chi-square test for categorical variables, along with Fisher’s exact test. Means and medians of maternal age were also compared. The following variables were compared between the case and referent populations: maternal age (categorical), maternal education, ethnicity, wealth quintile, ANC attendance, parity, previous stillbirth, antepartum hemorrhage, hypertensive disorder during pregnancy, medical disorder during pregnancy, multiple births, obstetric complications during labor, FHRM per protocol, use of partogram, mode of delivery, sex of baby, weight for gestational age at birth, gestational age of baby and a combination variable including both weight for gestational age and gestational age at birth.

Univariate logistic regression was used to determine the level of association between different demographic/obstetric characteristics and intrapartum stillbirth that showed a difference (*p* < 0.001) in the chi-square analysis, between the referent and case populations. Multivariate logistic regression analysis was then conducted to determine the level of association between the demographic or obstetric characteristics and intrapartum stillbirth for those with a significant association in the univariate model.

To the greatest extent possible, missing data was minimized; however, there were missing data for some background characteristics of the mothers. We used the multiple imputation method to deal with this data that was missing at random [35].

## Results

**Fig. 1:**
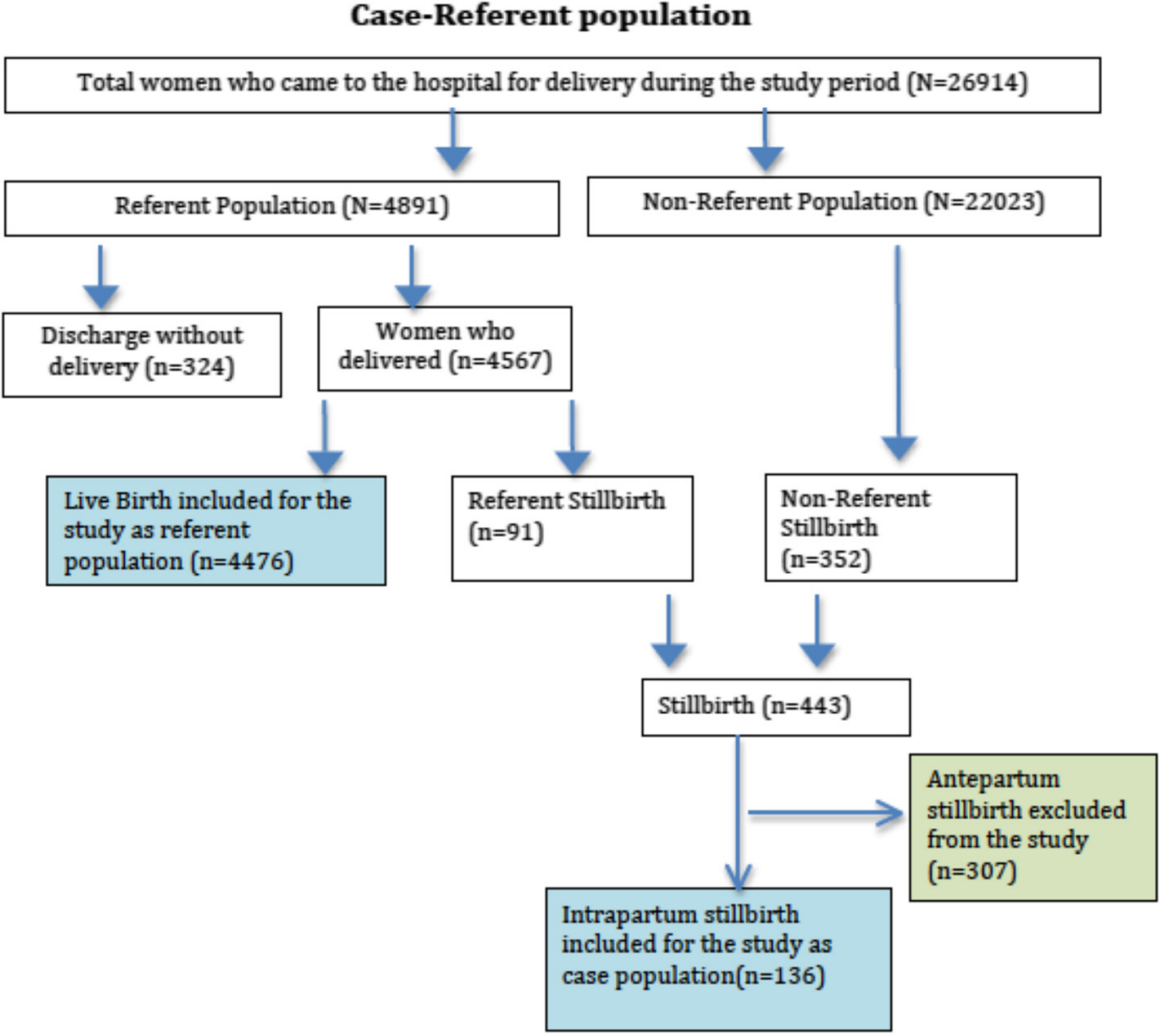
Flowchart of study population

During the study period a total of 26,914 women came to the hospital for delivery, of which 4,891 were selected to be in the referent population; however, of these, 324 mothers were discharged without delivering. Of the total referent population who delivered at the hospital, 4,476 infants were live-born and 91 were stillborn. Among the non-referent population there were 352 stillbirths. Thus, during the study period, 443 stillbirths occurred among the referent and non-referent populations combined, giving a stillbirth rate of 17.6 per thousand deliveries. Of the 443 stillbirths, 136 (30.7 %) were intrapartum stillbirths, giving an intrapartum stillbirth rate of 5.3 per thousand deliveries (Fig. 1). In a quarter of the observed deliveries, FHRM was performed according to protocol, whereas partogram use was per protocol in 50 % of deliveries.

Table 1 shows the demographic and obstetric characteristics of women in both the referent and case populations. The mean age of mothers in the case and referent populations were 25.7 and 23.7 years, respectively. The median age of mothers in the referent population was 23 versus 24 years in the case population. Differences between the two population groups were significant in the following categories: maternal age, education, wealth status, ANC, and parity. Significant differences were also seen between the case and referent populations in the presence of previous stillbirth, antepartum hemorrhage, hypertensive disorder during pregnancy, multiple birth, obstetric complication during labor, FHRM as per protocol, partogram use, mode of delivery, weight for gestational age, gestational age of the baby, and the combination of weight for gestational age and gestational age.

**Table 1:** Demographic and obstetric characteristics of referent women with live births and women with intrapartum stillbirth

Variable	Referent Live Birth (*N* = 4476)	Intrapartum Stillbirth (*N* = 136)	*P*-value*
Maternal age in years			
Mean ± SD	23.7 ± 4.4	25.7 ± 6.3	
Median (IQR)	23.0 (20–26)	24.0 (20–30)	
	n (%)	n (%)	
Maternal age (5-year interval)			
< 20	1224 (27.3)	34 (25.0)	*p* < 0.001
20–25	1957 (43.7)	45 (33.1)	
26–30	973 (21.7)	28 (20.6)	
> 30	322 (7.2)	29 (21.3)	
Maternal education			
Primary education (5 years) or less	1459 (32.6)	17 (12.5)	*p* < 0.001
Six years of education or more	3017 (67.4)	119 (87.5)	
Ethnicity			
Brahmin/Chhetri (hill or terai)	1733 (38.7)	42 (30.9)	*p* = 0.278
Relatively advantaged Janajatis	812 (18.1)	22 (16.2)	
Disadvantaged Janajatis	1293 (28.9)	48 (35.3)	
Non-Dalit (terai)	369 (8.2)	12 (8.8)	
Dalit (hill and terai)	235 (5.3)	11 (8.1)	
Muslim	34 (0.8)	1 (0.7)	
Wealth quintile			
Poorest	787 (19.0)	16 (34.0)	*p* < 0.01
Poorer	805 (19.5)	11 (23.4)	
Middle	864 (20.9)	7 (14.9)	
Richer	837 (20.2)	7 (14.9)	
Richest	845 (20.4)	6 (12.8)	
Antenatal Care Attendance			
At least one visit	3904 (87.2)	79 (58.1)	*p* < 0.001
No ANC	572 (12.8)	57 (41.9)	
Parity			
Primipara	2418 (54.0)	64 (47.1)	*p* < 0.001
Multipara (1–2)	1869 (41.8)	51 (37.5)	
Multipara (3 or more)	189 (4.2)	21 (15.4)	
Previous stillbirths			
No	4380 (97.9)	130 (95.6)	*p* = 0.08
Yes	96 (2.1)	6 (4.4)	
Antepartum hemorrhage			
No	4352 (97.2)	118 (86.8)	*p* < 0.001
Yes	124 (2.8)	18 (13.2)	
Hypertensive disorder during pregnancy			
No	4167 (93.1)	121 (89.0)	*p* = 0.05
Yes	309 (6.9)	15 (11.0)	
Medical disorder during pregnancy			
No	4262 (95.2)	127 (93.4)	*p* = 0.2
Yes	214 (4.8)	9 (6.6)	
Multiple birth			
No	4438 (99.2)	126 (92.6)	*p* < 0.001
Yes	38 (0.8)	10 (7.4)	
Obstetric complication during labor^a^			
No	3965 (88.6)	69 (50.7)	*p* < 0.001
Yes	511 (11.4)	67 (49.3)	
Fetal Heart Rate monitoring as per protocol			
Yes	1100 (24.6)	9 (6.6)	*p* < 0.001
No	3376 (75.4)	127 (93.4)	
Use of Partogram			
Yes	2272 (50.8)	15 (11.0)	*p* < 0.001
No	2204 (49.2)	121 (89.0)	
Mode of delivery			
Vaginal	3464 (77.4)	92 (67.6)	*p* = 0.007
C-section	1012 (22.6)	44 (32.4)	
Sex of newborn			
Female	2103 (47.0)	52 (38.2)	*p* = 0.45
Male	2373 (53.0)	84 (61.8)	
Weight per gestational age			
Appropriate-for-gestational age	2796 (62.5)	73 (53.7)	*p* < 0.02
Small-for-gestation age	1680 (37.5)	63 (46.3)	
Gestational age			
< 37 weeks	4113 (91.9)	74 (54.4)	*P* < 0.001
≥ 37 weeks	363 (8.1)	62 (45.6)	
Gestational age and weight per gestational age			
AGA and ≥ 37 weeks	2542 (56.8)	34 (25.0)	*p* < 0.001
SGA and ≥ 37 weeks	1604 (35.8)	40 (29.4)	
AGA and < 37 weeks	254 (5.7)	39 (28.7)	
SGA and < 37 weeks	76 (1.7)	23 (16.9)	

*p-value determined by Pearson’s chi-square test or Fisher’s exact test

^a^Obstetric complications during labor included: antepartum hemorrhage, hypertensive disorder, mal-presentation, prolonged labor, and cord prolapse

The univariate logistic regression analysis showed an association between the following risk factors and intrapartum stillbirth: increasing maternal age; being from the poorest wealth quintile; lack of ANC; increasing parity; incidence of antepartum hemorrhage or hypertensive disorder during pregnancy; having multiple births; the presence of obstetric complications during labor; inadequate adherence to FHRM protocol; non-use of the partogram during labor; delivery by cesarean section; having an infant who is SGA, an infant who was preterm, or an infant who was born both SGA and preterm (Table 2).

**Table 2:** Demographic and obstetric factors associated with intrapartum stillbirth

Variables*	Crude Odds Ratio* (95 % CI)	Adjusted Odds Ratio (95 % CI)
Maternal age (linear)	1.1 (1.0–1.1)	1.0 (0.9–1.1)
Maternal education		
Primary education or less	3.4 (2.0–5.7)	3.2 (1.8–5.5)
Second education or more	1	
Wealth quintile		
Non-poor	1	
Poor	1.6 (1.0–2.7)	1.8 (1.1–3.4)
Antenatal care		
At least one ANC visit	1	
No ANC	4.9 (3.5–7.0)	4.8 (3.2–7.2)
Parity		
Primi		
Multi-para	1.6 (1.4–1.8)	1.2 (1.0–1.5)
Antepartum hemorrhage		
No		
Yes	5.4 (3.2–9.1)	2.1 (1.1–4.2)
Hypertensive disorder during pregnancy		
No		
Yes	1.7 (1.0–2.9)	0.9 (0.5–1.8)
Multiple birth		
No		
Yes	9.3 (4.5–19.0)	3.0 (1.9–5.4)
Obstetric complication during intrapartum period		
No		
Yes	7.5 (5.3–10.7)	4.5 (2.9–6.9)
Fetal Heart Rate monitoring as per protocol		
Yes		
No	4.6 (2.3–9.1)	1.9 (1.5–2.4)
Use of partogram		
Yes		
No	8.3 (4.9–14.2)	2.1 (1.1–4.1)
Mode of delivery		
Vaginal		
Cesarean-section	1.6 (1.1–2.4)	0.8 (0.7–1.0)
Weight per gestational age		
Appropriate-for-gestational age	1.4 (1.0–2.0)	1.8 (1.2–1.7)
Small-for-gestation age		
Gestational age		
< 37 weeks	9.5 (6.7–13.5)	5.4 (3.5–8.2)
≥ 37 weeks		
Gestational age and weight per gestational age		
SGA and < 37 weeks	11.8 (7.1–19.5)	9.0 (7.3–15.5)
Others		

*Variables selected based on significant differences (*p* < 0.001) shown between the case and referent populations by Pearson’s chi-square test or Fisher’s exact test

a Crude odds ratio determined through univariate logistic regression analysis for likelihood of intrapartum stillbirth

Multivariate analysis was then performed with all significant (as determined by univariate analysis above) risk factors included in the multivariate model. Women with less education were three times more likely to have an intrapartum stillbirth as compared to those who had more education (AdjOR, 3.2 95 % CI-1.8–5.5). Women who did not attend any ANC checkups faced a five-fold increased risk for intrapartum stillbirth compared to those who went for at least one checkup (AdjOR, 4.8 95 % CI 3.2–7.2). Women from the poorest families were two times more likely to have an intrapartum stillbirth as compared to women from higher wealth quintiles (AdjOR 1.8, 95 % CI-1.1–3.4). Women who experienced antepartum hemorrhage had a two-fold higher risk of intrapartum stillbirth than women who did not (AdjOR 2.1, 95 % CI 1.1–4.2). Women who had multiple births were three times more likely to have an intrapartum stillbirth than women who had single births (Adj. OR-3.0, 95 % CI- 1.9–5.4). Women who had any obstetric complication during the labor period were four times more likely to experience intrapartum stillbirth (AdjOR-4.5, 95 % CI 2.9–6.9). Women whose FHRM was not done per protocol and those for whom a partogram was not used were two times more likely to have an intrapartum stillbirth (AdjOR-1.9, 95 % CI 1.5–2.4; and AdjOR-2.1, 95 % CI 1.1–4.1). Intrapartum stillbirths were two times more likely when the infant was SGA compared to AGA (AdjOR-1.8, 95 % CI-1.2–1.7). Intrapartum stillbirths were five times more likely to be delivered preterm than at a term gestational age (AdjOR-5.4, 95 % CI 3.5–8.2). Infants who were born preterm with a SGA had a nine-fold increased risk for intrapartum stillbirth compared to those delivered at term who were AGA (AdjOR-9.0, 95 % CI 7.3–15.5) (Table 2).

## Discussion

Our study examined various demographic and obstetric risk factors and their association with intrapartum stillbirth in a tertiary hospital setting in Nepal. Women who delivered a SGA infant prematurely had the highest risk for intrapartum stillbirth. Poor women with less education and poor utilization of ANC also had an increased risk for intrapartum stillbirth. Women who experienced antepartum hemorrhage, had multiple births or obstetric complications during labor also had a higher risk for intrapartum stillbirth. Finally, women who did not receive adequate care during the intrapartum period, i.e. sub-standard FHRM and lack of labor progression monitoring with a partogram, were also more likely to have had an intrapartum stillbirth.

Although findings from hospital-based studies may have limited generalizability at the population level, the lack of a periodic national perinatal health survey or a vital registration system in Nepal necessitates the use of hospital-based studies as one of the best available options to identify burden of disease in this setting. The information gathered from these studies is vital for the improvement of various clinical practices, including care given during the intrapartum period; additionally, the information can also be used to improve care at the community level, through the translation of best practice to these settings. One possible limitation of this study is the potential for under-reporting or a lack of reporting of maternal obstetric or medical conditions, especially in women facing the bereavement of delivering a stillborn infant. Similarly, not all women, even those who attended an ANC visit, received screening for medical and/or obstetric complications during pregnancy, so there could be an additional under-reporting of these conditions. Another limitation was the timing of enrollment of women in referent and case population. The women in referent population were enrolled at the time of admission while the women in case population were enrolled at the time of delivery. Another potential limitation is that the population-based reference standards used for defining birth weight for gestational age were not specific to Nepal, as no such standards exist; therefore, these reference standards were based on a U.S. population.

A study in Gambia by Ha et al. [15] showed a similar increase in the risk for intrapartum stillbirth among women whose families were subjected to socio-economic deprivation, potentially because poorer women receive sub-optimal pregnancy care. These women may not possess adequate resources to be able to afford the out-of-pocket expenses associated with screenings during pregnancy, and some may not even be able to access a skilled provider to receive an ANC checkup [15].

ANC plays a vital role in the maintenance or management of a woman’s health during pregnancy, and many women who lack access to ANC checkups are at an increased risk of intrapartum stillbirth. Our results are consistent with other studies done in Gambia and Zimbabwe [12, 36]. ANC provided by a skilled provider can help women to better understand the growth and development requirements of their fetus and the support it needs during this time, as well as to increase their awareness of the importance of maintaining an adequate health and nutritional status throughout pregnancy [37, 38]. ANC checkups also provide a platform for a discussion with women on how to prepare for delivery, identify potential danger signs during pregnancy and labor, and understand when it is time to seek care because delivery is imminent [39, 40]. Less educated women may have less access to and understanding of this valuable information, a lack of which has consistently been identified as a risk factor for intrapartum stillbirth in both high- and low-income countries [13, 41]. ANC checkups provide a valuable method for improving the understanding of mothers about pregnancy, birth preparedness, danger signs, and care seeking; and furthermore facilitate open discussion between the mother and her skilled provider.

Obstetric complications occurring during labor or delivery, including mal-presentation or prolonged labor, can cause detrimental, and potentially irreversible, insults to the fetus during the intrapartum period, potentially leading to death [11, 42]. These intrapartum stillbirths might have been averted if better obstetric care and fetal and maternal monitoring were available. In settings where FHRM is sub-optimal and partograms are not used to monitor labor progression, the risk of death further increases [43].

Multiple births have been associated with maternal morbidities, such as preterm labor and antepartum hemorrhage; in fact, the majority of twin pregnancies are associated with the delivery of SGA babies. Furthermore, in some cases, multiple births can lead to death of the fetus during the antepartum and intrapartum periods [44–46]. Studies have shown that women with multiple pregnancies who receive adequate obstetric care during pregnancy and the intrapartum period, have fewer adverse outcomes [47].

As shown in this study and several other previous studies, preterm delivery combined with having a SGA carries the highest risk for intrapartum stillbirth, with the risk of death increasing in very premature deliveries [41, 48, 49].

One of the key finding from our study is that sub-optimal use of FHRM and a partogram for the monitoring of labor progression are each associated with intrapartum stillbirth. A potential reason for this is that high-risk deliveries, such as those involving fetal distress, are not identified, which can lead to intrapartum insults on the fetus. Similarly, interventions required to prevent or manage prolonged labor, or other obstetric complications during labor, cannot be detected in a timely manner if a partogram is not used to monitor the progression of labor. Complications, such as prolonged labor, mal-presentation of the fetus, or maternal medical condition, can lead to severe fetal compromise during the intrapartum period and thus to intrapartum stillbirth, which might have been prevented in a tertiary care setting where emergency obstetric care is available.

Inadequate adherence to standardized protocols for intrapartum monitoring can be due to a multitude of factors. These factors may include: inadequate institutional leadership and/or support to improve clinical practice, shortages of staff, poor knowledge on the use of the partogram or FHRM, heavy workload for an inadequate number of staff, or inadequate understanding of the relevance and importance of the use of a partogram in preventing obstructed labor, as shown by studies in Africa [50–53]. Further research in this area is critical, and should explore potential contextual barriers preventing adherence to standard protocols, with the aim of identifying evidence-based interventions that facilitate improved adherence to these protocols.

## Conclusion

Our study has identified the combination of preterm birth and SGA as carrying the highest risk for intrapartum stillbirth, and highlights several other preventable risk factors associated with intrapartum stillbirth. In doing so, we have also highlighted the need for early implementation of adequate preventive strategies; including the proper use of FHRM and the partogram in order to better identify potential complications during the intrapartum period. As an effort to reduce the preventable burden of intrapartum stillbirth, the government of Nepal could use a two-pronged approach: first, efforts must be made to improve the quality of care provided during the antenatal and intrapartum periods, and second, conscious efforts are also needed to decrease the gap in equity and to promote an equitable distribution of antenatal and intrapartum healthcare services, so that poor and marginalized populations are not left behind. The losses that families face as a result of stillbirth have been recognized and target goals for the reduction of these losses have been set as part of the recent global agenda, with the aim to reduce the number of stillbirths to 10 or less per 1,000 deliveries by 2035. Adequate prevention and management of identified risk factors constitute major steps in the reduction of the number of intrapartum-related stillbirths.

## Abbreviations

AdjOR: Adjusted odds ratio
AGA: Appropriate for gestational age
ANC: Antenatal care attendance
FHRM: Fetal heart rate monitoring
SGA: Small-for-gestational age

## References

[1] Lawn JE, Blencowe H, Pattinson R, Cousens S, Kumar R, Ibiebele I, Gardosi J, Day LT, Stanton C (2011). Stillbirths: where? when? why? how to make the data count?. Lancet.

[2] Froen JF, Cacciatore J, McClure EM, Kuti O, Jokhio AH, Islam M, Shiffman J (2011). Stillbirths: why they matter. Lancet.

[3] GBD Mortality, Causes of Death C (2015). Global, regional, and national age-sex specific all-cause and cause-specific mortality for 240 causes of death, 1990–2013: a systematic analysis for the global burden of disease study 2013. Lancet.

[4] Cousens S, Blencowe H, Stanton C, Chou D, Ahmed S, Steinhardt L, Creanga AA, Tuncalp O, Balsara ZP, Gupta S, Say L, Lawn JE (2011). National, regional, and worldwide estimates of stillbirth rates in 2009 with trends since 1995: a systematic analysis. Lancet.

[5] Flenady V, Middleton P, Smith GC, Duke W, Erwich JJ, Khong TY, Neilson J, Ezzati M, Koopmans L, Ellwood D, Fretts R, Froen JF (2011). The lancet’s stillbirths series steering committee: stillbirths: the way forward in high-income countries. Lancet.

[6] Lawn JE, Lee AC, Kinney M, Sibley L, Carlo WA, Paul VK, Pattinson R, Darmstadt GL (2009). Two million intrapartum-related stillbirths and neonatal deaths: where, why, and what can be done?. Int J Gynaecol Obstet.

[7] Hofmeyr GJ, Haws RA, Bergstrom S, Lee AC, Okong P, Darmstadt GL, Mullany LC, Oo EK, Lawn JE (2009). Obstetric care in low-resource settings: what, who, and how to overcome challenges to scale up?. Int J Gynaecol Obstet.

[8] Bhutta ZA, Lassi ZS, Blanc A, Donnay F (2010). Linkages among reproductive health, maternal health, and perinatal outcomes. Semin Perinatol.

[9] Lawn JE, Yakoob MY, Haws RA, Soomro T, Darmstadt GL, Bhutta ZA (2009). 3.2 million stillbirths: epidemiology and overview of the evidence review. BMC Pregnancy Childbirth.

[10] Di Mario S, Say L, Lincetto O (2007). Risk factors for stillbirth in developing countries: a systematic review of the literature. Sex Transm Dis.

[11] Mmbaga BT, Lie RT, Olomi R, Mahande MJ, Olola O, Daltveit AK (2012). Causes of perinatal death at a tertiary care hospital in Northern Tanzania 2000–2010: a registry based study. BMC Pregnancy Childbirth.

[12] Jammeh A, Vangen S, Sundby J (2010). Stillbirths in rural hospitals in the gambia: a cross-sectional retrospective study. Obstet Gynecol Int.

[13] Nahar S, Rahman A, Nasreen HE (2013). Factors influencing stillbirth in bangladesh: a case–control study. Paediatr Perinat Epidemiol.

[14] McClure EM, Goldenberg RL (2009). Stillbirth in developing countries: a review of causes, risk factors and prevention strategies. J Matern Fetal Neonatal Med.

[15] Ha YP, Hurt LS, Tawiah-Agyemang C, Kirkwood BR, Edmond KM (2012). Effect of socioeconomic deprivation and health service utilisation on antepartum and intrapartum stillbirth: population cohort study from rural Ghana. PLoS one.

[16] Lawn J, Shibuya K, Stein C (2005). No cry at birth: global estimates of intrapartum stillbirths and intrapartum-related neonatal deaths. Bull World Health Organ.

[17] Wilkinson D (1995). Avoidable perinatal deaths in a rural hospital: strategies to improve quality of care. Trop Dr.

[18] Buchmann EJ, Pattinson RC, Nyathikazi N (2002). Intrapartum-related birth asphyxia in South Africa--lessons from the first national perinatal care survey. S Afr Med J.

[19] Pattinson RC (2003). Why babies die--a perinatal care survey of South Africa, 2000–2002. S Afr Med J.

[20] Hinderaker SG, Olsen BE, Bergsjo PB, Gasheka P, Lie RT, Havnen J, Kvale G (2003). Avoidable stillbirths and neonatal deaths in rural Tanzania. BJOG.

[21] Stanton C, Lawn JE, Rahman H, Wilczynska-Ketende K, Hill K (2006). Stillbirth rates: delivering estimates in 190 countries. Lancet.

[22] UNICEF (2015). Government of Nepal: Nepal multiple indicator cluster survey 2014.

[23] Ministry of Health and Population, New Era, ICF Macro, USAID (2011). Nepal demographic and health survey 2011.

[24] Ministry of Health and Population, GoN (2010). The Aama programme: an initiative towards reducing maternal and newborn deaths in Nepal.

[25] Powell-Jackson T, Hanson K (2012). Financial incentives for maternal health: impact of a national programme in Nepal. J Health Econ.

[26] Ashish KC, Malqvist M, Wrammert J, Verma S, Aryal DR, Clark R, Naresh PK, Vitrakoti R, Baral K, Ewald U (2012). Implementing a simplified neonatal resuscitation protocol-helping babies breathe at birth (HBB) - at a tertiary level hospital in Nepal for an increased perinatal survival. BMC Pediatr.

[27] Froen JF, Gordijn SJ, Abdel-Aleem H, Bergsjo P, Betran A, Duke CW, Fauveau V, Flenady V, Hinderaker SG, Hofmeyr GJ, Jokhio AH, Lawn J, Limbiganon P, Meriald M, Pattinson R, Shankar A (2009). Making stillbirths count, making numbers talk - issues in data collection for stillbirths. BMC Pregnancy Childbirth.

[28] World Bank and DFID (2006). Unequal citizens: gender, caste and ethnic exclusion in Nepal –summary report.

[29] Howe LD, Hargreaves JR, Gabrysch S, Huttly SR (2009). Is the wealth index a proxy for consumption expenditure? a systematic review. J Epidemiol Community Health.

[30] Rutsein SO, Johnson K (2004). The DHS wealth index- DHS comparative reports no. 6.

[31] Filmer D, Pritchett LH (2001). Estimating wealth effects without expenditure data--or tears: an application to educational enrollments in states of India. Demography.

[32] Opara EI, Zaidi J (2007). The interpretation and clinical application of the word ‘parity’: a survey. BJOG.

[33] World Health Organization: Managing complications in pregnancy and childbirth: a guide for midwives and doctors. In. Edited by WHO. 20 Avenue Appia, 1211 Geneva 27, Switzerland: World Health Organization; 2000.

[34] Alexander GR, Himes JH, Kaufman RB, Mor J, Kogan M (1996). A United States national reference for fetal growth. Obstet Gynecol.

[35] Barnard J, Meng XL (1999). Applications of multiple imputation in medical studies: from AIDS to NHANES. Stat Methods Med Res.

[36] Feresu SA, Harlow SD, Welch K, Gillespie BW (2005). Incidence of stillbirth and perinatal mortality and their associated factors among women delivering at Harare Maternity Hospital, Zimbabwe: a cross-sectional retrospective analysis. BMC Pregnancy Childbirth.

[37] Pervin J, Moran A, Rahman M, Razzaque A, Sibley L, Streatfield PK, Reichenbach LJ, Koblinsky M, Hruschka D, Rahman A (2012). Association of antenatal care with facility delivery and perinatal survival - a population-based study in Bangladesh. BMC Pregnancy Childbirth.

[38] Rahman A, Moran A, Pervin J, Rahman A, Rahman M, Yeasmin S, Begum H, Rashid H, Yunus M, Hruschka D, Arifeen SE, Streatfield PK, Sibley L, Bhuiya A, Koblinsky M (2011). Effectiveness of an integrated approach to reduce perinatal mortality: recent experiences from Matlab Bangladesh. BMC Public Health.

[39] McClure EM, Pasha O, Goudar SS, Chomba E, Garces A, Tshefu A, Althabe F, Esamai F, Patel A, Wright LL, Moore J, Bhalchandra KS, Belizan JM, Saleem S, Derman RJ, Carlo WA, Hambidge MK, Buekens P, Licehty EA, Bose C, Thomas MK, Jobe AH, Goldenberg RL, Global Network Investigators (2011). Epidemiology of stillbirth in low-middle income countries: a global network study. Acta Obstet Gynecol Scand.

[40] Ca K, Nelin V, Wrammert J, Ewald U, Vitrakoti R, Baral GN, Malqvist M (2015). Risk factors for antepartum stillbirth: a case–control study in Nepal. BMC Pregnancy Childbirth.

[41] Getahun D, Ananth CV, Kinzler WL (2007). Risk factors for antepartum and intrapartum stillbirth: a population-based study. Am J Obstet Gynecol.

[42] Baqui AH, Choi Y, Williams EK, Arifeen SE, Mannan I, Darmstadt GL, Black RE (2011). Levels, timing, and etiology of stillbirths in Sylhet district of Bangladesh. BMC Pregnancy Childbirth.

[43] Goldenberg RL, McClure EM, Kodkany B, Wembodinga G, Pasha O, Esamai F, Tshefu A, Patel A, Mabaye H, Goudar S, Saleem S, Waikar M, Langer A, Bose CL, Rubens CE, Wright LL, Moore J, Blanc A (2013). A multi-country study of the “intrapartum stillbirth and early neonatal death indicator” in hospitals in low-resource settings. Int J Gynaecol Obstet.

[44] Mutihir JT, Pam VC (2007). Obstetric outcome of twin pregnancies in Jos, Nigeria. Niger J Clin Pract.

[45] Aisien AO, Olarewaju RS, Imade GE (2000). Twins in Jos Nigeria: a seven-year retrospective study. Med Sci Monit.

[46] Fakeye O (1986). Perinatal factors in twin mortality in Nigeria. Int J Gynaecol Obstet.

[47] Akaba GO, Agida TE, Onafowokan O, Offiong RA, Adewole ND (2013). Review of twin pregnancies in a tertiary hospital in Abuja, Nigeria. J Health Popul Nutr.

[48] Engmann C, Matendo R, Kinoshita R, Ditekemena J, Moore J, Goldenberg RL, Tshefu A, Carlo WA, McClure EM, Bose C, Wright LL (2009). Stillbirth and early neonatal mortality in rural Central Africa. Int J Gynaecol Obstet.

[49] Lee AC, Mullany LC, Tielsch JM, Katz J, Khatry SK, Leclerq SC, Adhikari RK, Darmstadt GL (2011). Community-based stillbirth rates and risk factors in rural Sarlahi Nepal. Int J Gynaecol Obstet.

[50] Kawuwa MB, Mairiga AG, Usman HA (2006). Maternal mortality: barriers to care at the health facility--health workers perspective. J Obstet Gynaecol.

[51] Yisma E, Dessalegn B, Astatkie A, Fesseha N (2013). Knowledge and utilization of partograph among obstetric care givers in public health institutions of Addis Ababa Ethiopia. BMC Pregnancy Childbirth.

[52] Opiah MM, Ofi AB, Essien EJ, Monjok E (2012). Knowledge and utilization of the partograph among midwives in the Niger Delta Region of Nigeria. Afr J Reprod Health.

[53] Fawole AO, Hunyinbo KI, Adekanle DA (2008). Knowledge and utilization of the partograph among obstetric care givers in south west Nigeria. Afr J Reprod Health.

